# Directional selection for flowering time leads to adaptive evolution in *Raphanus raphanistrum* (Wild radish)

**DOI:** 10.1111/eva.12350

**Published:** 2016-02-17

**Authors:** Michael B. Ashworth, Michael J. Walsh, Ken C. Flower, Martin M. Vila‐Aiub, Stephen B. Powles

**Affiliations:** ^1^ Australian Herbicide Resistance InitiativeSchool of Plant BiologyThe University of Western AustraliaCrawleyWAAustralia; ^2^ Department of Agriculture and EnvironmentSchool of ScienceCurtin UniversityBentleyWAAustralia; ^3^ School of Plant BiologyThe University of Western AustraliaCrawleyWAAustralia; ^4^ IFEVA‐CONICETFacultad de AgronomíaUniversidad de Buenos AiresBuenos AiresArgentina

**Keywords:** biomass, evolution, flowering height, flowering time, phenotypic resistance, wild radish

## Abstract

Herbicides have been the primary tool for controlling large populations of yield depleting weeds from agro‐ecosystems, resulting in the evolution of widespread herbicide resistance. In response, nonherbicidal techniques have been developed which intercept weed seeds at harvest before they enter the soil seed bank. However, the efficiency of these techniques allows an intense selection for any trait that enables weeds to evade collection, with early‐flowering ecotypes considered likely to result in early seed shedding. Using a field‐collected wild radish population, five recurrent generations were selected for early maturity and three generations for late maturity. Phenology associated with flowering time and growth traits were measured. Our results demonstrate the adaptive capacity of wild radish to halve its time to flowering following five generations of early‐flowering selection. Early‐maturing phenotypes had reduced height and biomass at maturity, leading to less competitive, more prostrate growth forms. Following three generations of late‐flowering selection, wild radish doubled its time to flowering time leading to increased biomass and flowering height at maturity. This study demonstrates the potential for the rapid evolution in growth traits in response to highly effective seed collection techniques that imposed a selection on weed populations within agro‐ecosystems at harvest.

## Introduction

Agro‐ecosystems are productive environments placed under intense disturbance (Grime 1977). Despite this disturbance, genetically diverse weed species exhibit ruderal strategies that enable them to colonize, establish and successfully persist despite efforts to eradicate them (Grime 1977; Harper 1977). Recurrent use of chemical (herbicides), physical (cultivation) and cultural (agronomy) techniques allows intense selection on the life history, phenological and growth traits of plants (Mortimer 1997). For example, herbicide selection often results in the evolution herbicide resistance (Powles and Yu 2010) and adaptive changes in the timing of seed germination and seedling emergence (Kleemann and Gill 2013; Owen et al. 2014).

Herbicides for weed control are the dominant and most intensive selective force used in modern agriculture, resulting in the widespread evolution of herbicide resistance in 246 weed species worldwide (Heap 2015). However, with few new herbicide modes of action (Duke 2012) and the loss of available herbicides through regulation or the evolution of herbicide resistance, it has become necessary to develop new nonherbicidal weed control strategies (Murphy et al. 1998; Madafiglio et al. 2006; Walsh and Powles 2007). The most prominent of these are a range of techniques which intercept weed seeds at harvest before they re‐enter the soil seed bank (techniques collectively termed harvest weed seed control). These agricultural techniques have been reviewed (Walsh et al. 2012, 2013; Walsh and Powles 2014a,b) and are now being employed over large areas in Australia as well as being investigated for use in other grain‐growing nations.

Seed dispersal and seed return to the soil seed bank are key factors in the persistence of weed populations (Fernandez‐Quintanilla 1988). The flowering time of many weed species is synchronized with crop flowering (Tremblay and Colasanti 2007), so weeds often mature concurrently with crops. Consequently, grain harvesting techniques effectively intercept and redistribute weed seeds back onto the soil surface, replenishing the soil weed seed bank (Walsh et al. 2013). Intercepting and destroying weed seeds of annual weed species is a new technique to manage weeds in agro‐ecosystems (Walsh et al. 2013).

It is likely that weeds can adapt to any selective force (Jordan and Jannink 1997). Harvest weed seed control is a selective force favouring any mechanism that will enable plants to evade harvest interception. The efficacy of harvest weed seed control is contingent upon weed seeds being collected during the harvesting process, which is dependent upon the amount of weed seed retained on standing plants at crop harvest (Walsh and Powles 2014a,b). More prostrate forms (Ferris 2007) and/or earlier‐seed shedding phenotypes may evade harvest collection (Baker 1974). The selection of earlier‐flowering ecotypes is likely to increase the risk of seed/fruit abscission prior to harvest, resulting in harvest weed seed control evasion (Panetta et al. 1988).

*Raphanus raphanistrum* (wild radish) is among the worst weeds in global agriculture (Snow and Campbell 2005). In Australia, wild radish is considered to be the most problematic dicotyledonous weed species (Alemseged et al. 2001), causing significant yield losses in grain and horticultural crops (Code and Donaldson 1996; Blackshaw et al. 2002). Wild radish exhibits sufficient standing genetic variation (Conner et al. 2003; Madhou et al. 2005) to enable adaptive resistance evolution to multiple herbicide chemical classes (Hashem et al. 2001; Walsh et al. 2004a,b; Ashworth et al. 2014).

As approximately 95% of wild radish seed production is retained on the parent plant at harvest, harvest weed seed collection is ideal for controlling wild radish populations (Walsh and Powles 2014a,b). Currently, wild radish flowering time is synchronized with dryland field crops in Mediterranean climates. However, with the significant phenotypic variability in flowering time evident both within and between wild radish populations (Kercher and Conner 1996; Conner et al. 2003; Madhou et al. 2005), it is speculated that persistent collection of wild radish seed collection at crop harvest could impose a selection for early flowering time. The selection of earlier‐flowering phenotypes would likely result in the evolution of wild radish populations that display a shorter life cycle, allowing plants to set and shed seed prior to crop harvest (i.e. crop maturity). This study investigated the potential for recurrent directional selection to result in heritable changes in flowering time and fitness traits in wild radish.

## Materials and methods

### Plant material

**Figure 1:**
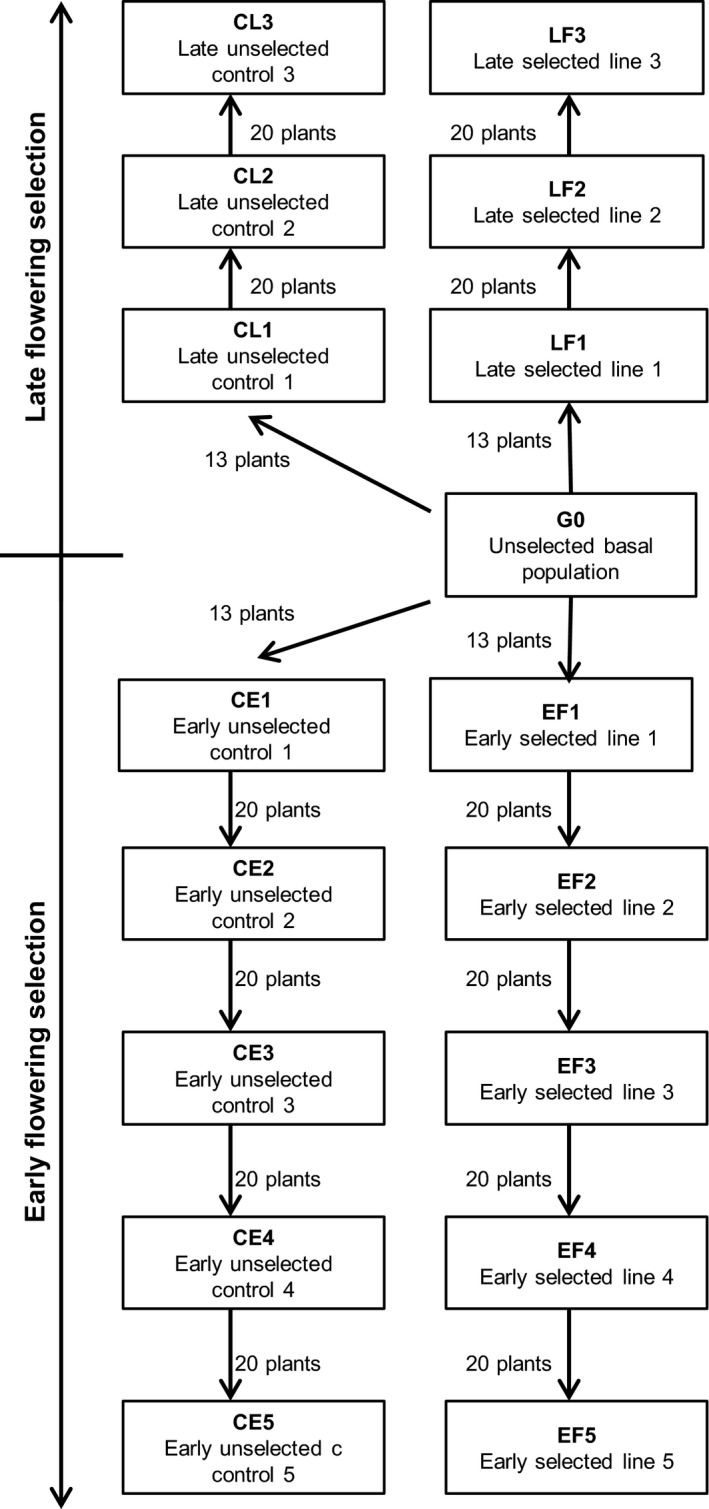
Hierarchy of flowering time selection applied to the commencing wild radish population (G0).

This selection study was conducted using a wild radish population (WARR7, referred hereafter as G0), originally collected in 1999 from Yuna, Western Australia (WA) (28.34°S, 115.01°E). This population has never been exposed to selection by herbicides or agronomic practices such as weed seed collection at harvest (Walsh et al. 2004a). Since collection, seed stocks of this herbicide susceptible population have been maintained and multiplied, ensuring no cross‐pollination with other populations to maintain its susceptibility. Commencing with this population, (G0), five successive generations of recurrent early‐flowering time (FT) selection was conducted in October 2011 (EF1), December 2011 (EF2), March 2012 (EF3), July 2012 (EF4) and December 2012 (EF5). Concurrently, three generations of late‐FT selection were conducted in September 2012 (LF1), January 2013 (LF2) and April 2013 (LF3). During each selection, control populations were maintained without selection in the same experimental conditions, except for the absence of FT selection (CE1–CE5; CL1–CL3) (Fig. 1). At all times, plants were well watered with optimum fertilization.

### Initial flowering date selection procedure

The initial FT selection (EF1) was made from a starting G0 population of 1300 plants (Table 1). Wild radish seeds (G0) greater than 2.2 mm in diameter were pregerminated on agar‐solidified water (0.6% w/v), at room temperature (20°C), in darkness for 2 days. Seeds with >5 mm of emerged radicle were transplanted (5 seedlings per pot) to a depth of 10 mm into 260 pots of 305 mm diameter, containing standard potting mixture (25% peat moss, 25% sand and 50% mulched pine bark). Pots were maintained in the outdoor growth facility at The University of Western Australia (Perth) during their normal winter–spring growing season (June–October). All pots were watered regularly and fertilized weekly with 2 g Scotts Cal‐Mg grower plus^™^ soluble fertilizer (N 15% [urea 11.6%, ammonium 1.4%, nitrate 2%], P 2.2%, K 12.4%, Ca 5%, Mg 1.8%, S 3.8%, Fe 120 mg kg^−1^, Mn 60 mg kg^−1^, Zn 15 mg kg^−1^, Cu 15 mg kg^−1^, B 20 mg kg^−1^, Mo 10 mg kg^−1^). The plants were monitored daily to observe the first signs of anthesis. At this time of first flowering, 13 plants (1%) were selected, based on the number of days from emergence to opening of the first flower (as marked by the protrusion of the corolla beyond the calyx). These selected plants were isolated to ensure cross‐pollination only among the selection and to prevent ingress of foreign pollen. Once all selected plants were flowering, all earlier flowers were removed to minimize any unintended drift in selection due to differences in female fitness among selected individuals (Sahli and Conner 2011). Newly opened flowers were crossed using the ‘Beestick method’, where a bee carcass was used to cross‐fertilize flowers as outlined by Williams (1980), ensuring a random pattern of cross‐pollination (panmixia), where each flower was randomly crossed with a flower from a different plant. At maturity, the same number of siliques were harvested from each plant in the population and bulked. Mature siliques were then processed using a modified ‘grist mill’ with seed progeny representing the first selected generation (EF1; LF1) (Table 1). Concurrently, a random sample of 13 plants were selected and maintained as described above to form the first generation of the unselected control line (CE1; CL1) (Table 1).

**Table 1:** Flowering time advancement [days to flowering and cumulative growing degree‐days (GDD)] of each wild radish accession during selection

	Selected generations						Unselected control generations			Phenotypic advancement in first flowering between selected and control generations	
	Selected line	Selection coefficient (1‐selected plants ratio)	Sowing date	Selection dates	Days to first flowering	Cumulative GDD (°C d)	Control line	Days to first flowering	Cumulative GDD (°C d)	Change in days to first flowering	Change in cumulative GDD (°C d)
Early‐flowering time selection	Commencing G0	0.99	5 Sept 2011	5 Oct 2011	30	446					
	EF1	0.95	9 Dec 2011	30 Dec 2011	21	382	CE1	26	467	−5	−85
	EF2	0.95	9 March 2012	31 March 2012	22	369	CE2	30	468	−8	−99
	EF3	0.95	23 May 2012	4 July 2012	42	349	CE3	72	521	−30	−172
	EF4	0.95	4 Dec 2012	24 Dec 2012	19	269	CE4	34	506	−15	−237
	EF5			Final early selected			CE5	Final early control		–	–
Late‐flowering time selection	Commencing G0	0.99	4 June 2012	4 Sept 2012	68 (92)^a^	447 (936)^b^					
	LF1	0.95	8 Nov 2012	6 Jan 2013	25 (48)^a^	512 (1024)^b^	CL1	21	482	4	30
	LF2	0.95	11 Feb 2013	14 April 2013	28 (62)^a^	484 (2214)^b^	CL2	21	436	7	48
	LF3			Final late selected			CL3	Final late control		–	–

a Bracketed data denotes days to last flowering selection (last 10% of the population).

b Bracketed data denotes cumulative degree‐days until final last flowering individuals were selected (last 10% of the population).

The initial FT selection for late flowering (LF1) was made from a commencing population of 1300 plants using the previously described procedure. All plants were monitored daily to observe anthesis. Only the last 13 plants to flower among the 1300 plants (1%) were selected. These plants were isolated and cross‐pollinated among themselves as previously described. At maturity, siliques were harvested and processed, with seed progeny representing the first long flowering selected generation (LF1) (Table 1).

### Subsequent selections general procedure

Subsequent directional FT selections were conducted in a temperature‐controlled glasshouse with natural light, where cooling was initiated above 25°C day and 15°C night. Large seed (>2.2 mm diameter) of the initial selected populations (EF1 or LF1) and the initial control populations (CE1 or CL1) were germinated on solidified water agar (0.6% w/v), at room temperature (20°C) in darkness for 2 days. After germination, 250 pregerminated seeds (>5 mm emerged radicle) were seeded into separate 220‐mm‐diameter pots, watered twice daily to field capacity using an automated irrigation system and fertilized weekly as previously described. The date of emergence was noted for each pot. At first flowering, 20 plants were selected from the 250 individuals based on the number of days from emergence to the opening of the first flower. These selected plants were isolated and crossed as previously described to produce early‐selected generations (EF2–EF5). Using this methodology, the late‐selected generations were also selected (LF2; LF3) (Fig. 1). Concurrently, 20 randomly selected seeds from each respective control line (CE1; CL1) were planted, maintained and crossed as previously described to produce unselected early‐control generations (CE2–CE5) and late‐control generations (CL2; CL3) (Fig. 1).

### Analysis of selection and crossing lines

The rate of FT progression was evaluated by growing the G0, selected (EF1–EF5; LF1–LF3) and control (CE1–CE5; CL1–CL5) generations at the same time within temperature‐regulated glasshouse conditions during a period of stable to gradually increasing day length (June onwards, 2013) (Supporting information). Large seed (>2.0 mm in diameter) from each population was pregerminated on agar (0.6% w/v)‐solidified water in darkness for 2 days. Seventy‐five seeds from each population (with >5 mm emerged radicle) were seeded 10 mm deep into individual 220‐mm‐diameter pots containing standard potting mixture (25% peat moss, 25% sand and 50% mulched pine bark). All selected, control and progeny generations were arranged within the glasshouse in a randomized block design (3 blocks of 25 plants per treatment) with the date of emergence noted for each pot. All pots were watered to field capacity every 2 h (during the day) using an automated irrigation system (Supporting information), with 2 g Scotts Cal‐Mag grower plus^™^ soluble fertilizer applied weekly, as previously described. For the duration of the experiment, temperatures were maintained at temperatures of 25°C day and 15°C night, above the base temperature for wild radish growth (4.5°C) (Reeves et al. 1981). Air temperature and daylight was recorded every 15 min using an environment‐controlling thermistor and light photometer (Schneider Electric; www.schneider-electric.com) located 1 m above the pots in the centre of the glasshouse. For the duration of the experiment, the date of flowering and height of the first flower were recorded daily for each individual. Above‐ground biomass at the initiation of flowering was cut and dried at 65°C for 7 days before weighing.

### Data analysis

To compare the FT response of recurrently selected wild radish populations, nonlinear regression analysis was performed using the DRC package in R 3.0.0 (R Development Core Team 2011; http://www.R-project.org) (Streibig et al. 1993). The observed population flowering over time was fitted to a four‐parameter logistic model (1):

(1) $$Y=\frac{c+(d-c)}{1+e^{b(\log x-\log e)}}$$

where *Y* denotes cumulative flowering as a percentage of the total population, *e* is the FD_50_ denoting the time or accumulated temperature to flowering response is half‐way between the upper limit, *d* (fixed to the total percentage of the population collected) and *c* the lower asymptotic value of *Y* (set to 0). The parameter *b* denotes the relative slope around *e*. FD_50_ parameter was compared between selected and unselected (G0) populations using the selectivity indices (SI) function (R 3.0.0) which determines whether the ratios between the FD_50_ values are significantly different (*P *<* *0.05). A lack‐of‐fit test was also applied to each curve to ascertain the appropriateness of the model [1] in R3.0.0.

The experiments were conducted at different times of the year; therefore, the different selections were compared using growing degree‐days (GDD) to flowering, as described by Marcellos and Single (1971) and equation (2):

(2) $$\text{GDD}=\frac{\sum (T_{\max}+T_{\min})}{2}-T_{\mathrm{base}}$$

where *T*_max_ is the daily maximum temperature, *T*_min_ is the daily minimum temperature and *T*_base_ is the base temperature for wild radish (4.5°C) (Reeves et al. 1981). During this study, it is assumed that *T*_base_ remained constant. Height of the first flower and above‐ground biomass at flowering were checked for homogeneity of variance, normality and independence of residuals as described by Onofri et al. (2010). The flowering time selections were then compared for these response variables using a one‐way analysis of variance (anova) in Genstat version 6.1.0.200 (VSN International, www.vsni.co.uk/genstat). Above‐ground biomass at flowering was log_10_‐transformed prior to a two‐way anova. Means were estimated and separated using Tukey's protected LSD at the 5% level of significance. Biomass data were back‐transformed prior to plotting. The relationship between the response variables (height of first flower and above‐ground biomass) and days to flowering was plotted using SigmaPlot v.12 (Systat Software Inc., San Jose, CA, USA).

## Results

### Effect of recurrent early‐flowering time selection

**Figure 2:**
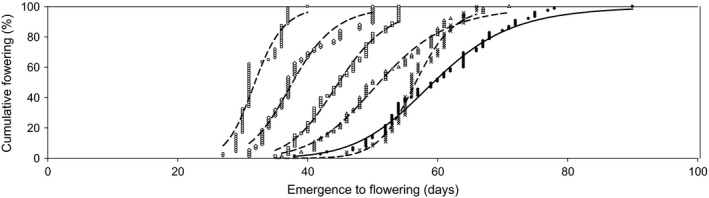
The observed population response to early‐flowering time selection against the unselected commencing wild radish population G0 (__•__). Early‐flowering time‐selected generations EF1 (…∆…), EF2 (…×…), EF3 (…□…), EF4 (…◊…) and EF5 (…○…). Each symbol represents cumulative data points of 75 replicate plants. The plotted lines are predicted cumulative flowering date curves fitted to a four‐parameter logistic model [1].

In one large final experiment, the G0 population and all successive selected (EF1–EF5; LF1–LF3) and the unselected control (CE1–CE5; CL1–CL3) populations were grown in a temperature‐controlled glasshouse to evaluate the population phenotypic change of each FT selection. Analysis of all accessions showed that FT was halved at the population level (FD_50_), following five successive generations of early‐FT selection. The FD_50_ parameter is the median time for the population to initiate its first flower. Early‐FT selection reduced the time from emergence to flowering from 59 days after emergence (DAE) (G0) to 29 DAE (EF5) (Fig. 2), reducing the thermal time requirement prior to flowering (GDD) from 634°C d (G0) to 344°C d (EF5) (see Supporting information). This reduction in FT was evident during each selection with thermal time requirement to flowering decreasing by 85, 99, 172 and 237°C d in the EF1 to EF4 generations, respectively, when compared to the concurrently grown but unselected controls (CE1–CE4) (Table 1). In the absence of selection, the control generations (CE1–CE5) changed FT negligibly compared with the G0 population, demonstrating that FT reductions in the selected generations (EF1–EF5) were primarily due to the effects of FT selection (Supporting information).

Flowering time reductions at the population level (FD_50_) in the early‐FT‐selected generations were found to be a result of a reduction in the distribution rather than any shift towards an earlier initiation of flowering. Following five generations of early‐FT selection, the initiation of flowering decreased by 11 days (EF5) (Fig. 2); however, the distribution of FT in the population decreased fourfold, from an initial range of 52 days (G0) to 13 days (EF5) (Fig. 2). This decrease in the distribution of FT resulted in 77% of the EF5 generation flowering prior to the initiation of flowering in the unselected G0 population.

**Figure 3:**
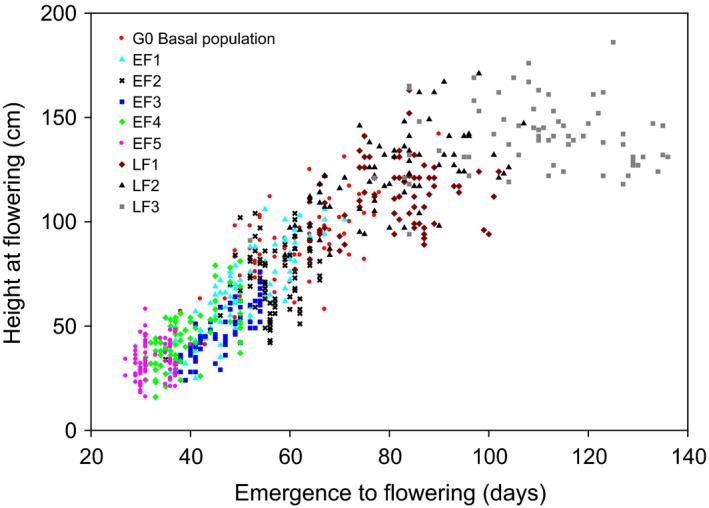
The relationship between the height of the first flower and days to flowering for the commencing (G0), early‐selected (EF1–EF5) and late‐selected (LF1–LF3) generations. Each symbol represents individual plants (each population *n *=* *75; LF3 *n *=* *64).

**Figure 4:**
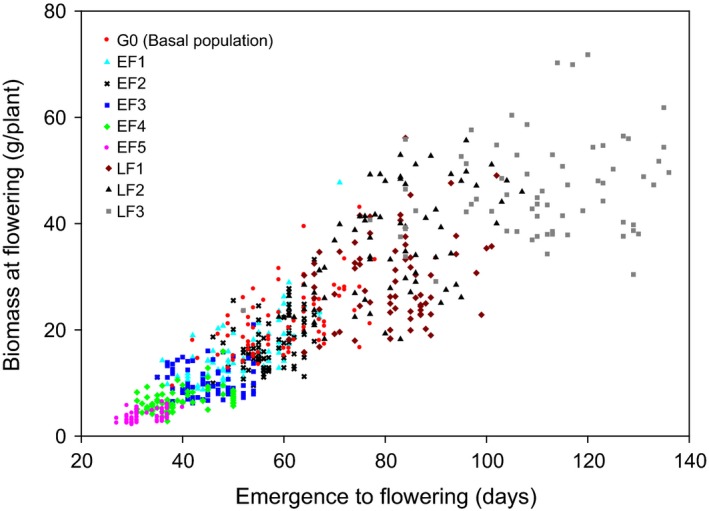
The relationship between biomass at flowering and days to flowering for the commencing (G0), early‐selected (EF1–EF5) and late‐selected (LF1–LF3) generations. Each symbol represents individual plant biomass measurements (each population *n* = 75; LF3 *n* = 64).

As well as reductions in FT, selection also led to reduced plant height and above‐ground biomass at flowering. Five generations of early‐FT selection reduced mean height at the initiation of flowering 2.6‐fold from 88 cm in the G0 population to 33 cm (EF5) (*P *<* *0.001) (Table 2). Concurrently, mean plant biomass at the initiation of flowering decreased 5.5‐fold from 22 g (G0) to 4 g (EF5) (*P *<* *0.001) (Table 2). In the absence of selection (CE1–CE5), there was no significant change in population biomass or flowering height from the G0 population (*P *>* *0.05) (Table 2). Both height and biomass at flowering decreased in plants that flowered earlier (Figs 3 and 4, respectively).

**Table 2:** Mean height of first flower and aboveground biomass at flowering for the commencing (G0), early‐selected (EF1–EF5) and late‐selected (LF1–LF3) generations

Selection	Selected line	Height (cm)	Biomass (g plant^−1^)
Early‐flowering time selection	EF5	33 a^a^	4 a
	EF4	44 b	7 ab
	EF3	46 b	10 b
	EF2	72 c	17 c
	EF1	69 c	16 c
Unselected control early flowering	CE5	90 e	22 d
	CE4	83 de	22 d
	CE3	89 de	21 d
	CE2	80 d	20 d
	CE1	88 de	21 d
Unselected	Commencing G0	88 de	22 d
Unselected control late flowering	CL1	84 de	20 d
	CL2	90 e	22 d
	CL3	87 de	19 d
Late‐flowering time selection	LF1	112 f	29 e
	LF2	121 f	35 f
	LF3	140 g	46 g

a Different letters indicate significant difference between means (Tukey separation) at *P* ≤ 0.05.

### Effect of recurrent late‐flowering time selection

**Figure 5:**
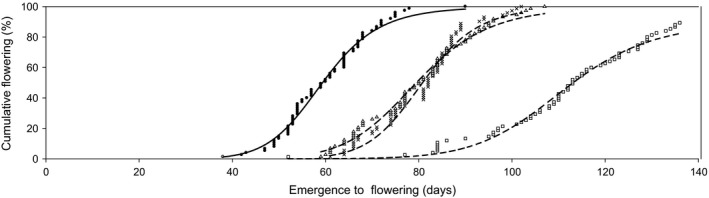
The observed population response to late‐flowering time selection against the unselected commencing wild radish population G0 (__•__). late‐selected generations LF1 (…×…), LF2 (…∆…) and LF3 (…□…). each symbol represents cumulative data points of 75 replicate plants [except LF3 = 65 plants (max 86%)]. the plotted lines are predicted cumulative flowering date curves fitted to a four‐parameter logistic model [1].

Conversely, late‐FT selection resulted in large stepwise increases in FT in both the first (LF1) and third (LF3) generation (Fig. 5). Following three generations of late‐FT selection, the length of the vegetative stage was doubled, from 59 DAE (G0; FD_50_) to 114 DAE (LF3; FD_50_) (Table 1), corresponding to a 2.1‐fold increase in the thermal time requirement prior to flowering (634°C d (G0) to 1314°C d (LF3) (Supporting information). The initiation of flowering was delayed by 23 days following a single generation of FT selection (LF1). Subsequent selections did not further delay the initiation of flowering (LF2; LF3) (Supporting information). Additional selections, however, progressively increased the distribution of flowering from 52 days in the G0 population to 84 days following three generations of late‐FT selection (Fig. 5). In the absence of selection, the concurrently grown generations (CL1–CL3) were not different from the G0 population (*P* > 0.05), again demonstrating that FT increases were primarily due to the effects of selection (Supporting information).

Late‐FT selection progressively increased plant biomass and height of the first flower. The height of the first flower progressively increased from 88 cm in the G0 population to 112 cm, 121 cm and 141 cm in the LF1, LF2 and LF3 generations, respectively (Table 2). Late‐FT selection also increased mean plant biomass at flowering from 22 g (G0) to 29 g, 35 g and 46 g per plant in the LF1, LF2 and LF3 generations, respectively (Table 2).

## Discussion

Sustainable agriculture is based on achieving a balance between producing highly productive and profitable crops whilst minimizing cost and energy inputs (Gomiero et al. 2011). The evolution of herbicide resistance in weed species infesting crops poses a significant threat to crop production (Powles and Yu 2010). As a consequence, there is an increased interest in nonchemical weed management tools (Walsh and Powles 2014a,b). However, over‐reliance of any single agronomic weed control practice is expected to result in rapid selection of adaptive traits, selected from the standing genetic variation within weed populations (Powles and Yu 2010).

The results of this study demonstrate that wild radish exhibits significant standing genetic variability to adapt to flowering time (FT) selection. Following five generations of early‐FT selection, wild radish FT (FD_50_) was halved, whilst three generations of late‐FT selection doubled FT at the population level. Bidirectional selection resulted in a total FT divergence of 83 days at the population level following five early and three late generations of flowering selection. The rapid FT response in this study is consistent with previous bidirectional FT selection results in studies of Chinese daikon radish (*Raphanus sativus* L.). These studies hinted that the nature of the genetic control of FT was likely to be polygenic with incomplete dominance (Vahidy 1969). Similar flowering time shifts in response to early‐FT selection has also been observed in both field and glasshouse environments in other closely related Brassica species such as wild mustard (*Brassica rapa* L.) (Franke et al. 2006; Franks et al. 2007; Franks 2011).

### Adaptability of wild radish

Wild radish is a genetically diverse, highly adaptable species which has been found to consistently thrive in a diverse range of environments (Madhou et al. 2005; Snow and Campbell 2005) and production systems (Alemseged et al. 2001; Borger et al. 2012). This study shows that when selected for reduced time to initiate flowering, wild radish plants can flower at far lower thermal requirements than normally observed in field populations. Previous studies have indicated that wild radish can reach anthesis in as little as 600°C d (Reeves et al. 1981; Cheam 1986; Malik et al. 2010). However, following five generations of early‐FT selection in this study, wild radish reduced its thermal requirement to 344°C d [FD_50_] at the population level. At an individual level, a thermal requirement of just 281°C d was observed resulting in a wild radish plant flowering 22 days after emergence (EF5) with a biomass of just 2.4 g. Identification of these early‐flowering individuals in the EF5 generation is significant as they became less sensitive to photoperiod or temperature cues, flowering under a short photoperiod of 9.5 h per day, at an average daily temperature of 15°C. Conversely, late‐FT selection over three generations increased thermal time to 1314°C d [FD_50_]. This study, however, understates the full adaptive response of wild radish to late‐FT selection, as the analysis of the selected populations was suspended at 149 DAE (or after 1565°C d) with 14% (11 plants) from the final LF3 generation still failing to initiate flowering. A total FT divergence of 127 days from the initiation of the first flowering individual to the suspension of this study is a clear demonstration of the remarkable capacity of wild radish populations to adapt FT when selected.

The genetic basis for FT adaptation in this study has not been determined; however, quantitative trait loci (QTL) studies in wild mustard, oilseed canola (*Brassica napus* L.) and *Arabidopsis thaliana* L. have identified that multiple loci are involved in flowering initiation in *A. thaliana* L (Osborn et al. 1997; Cai et al. 2008; Colautti and Barrett 2010; Raman et al. 2013). Over 80 different genes have been identified to affect FT initiation in response to external and endogenous cues (Simpson and Dean 2002). The progressive early‐FT shifts in this study are compatible with the polygenic accumulation of minor gene traits' as other hypotheses including variation in gene editing are possible. This study is also consistent with Vahidy (1969) and Conner (1993), who observed large shifts in phenology with late‐FT selection.

### Ecological and evolutionary significance and implications for weed management in agro‐ecosystems

The results of this study demonstrate the capacity of wild radish to adapt both phenologically and through growth traits. The selection treatments in this study mimic a strong selection force acting against plants that usually have synchronous flowering with field crops (i.e. harvest weed seed control). Recurrent selection for early flowering over five generations resulted in a halving of the flowering time (FD_50_) as well as a fourfold reduction in the distribution of flowering, resulting in 77% of individuals flowering before the initiation of flowering in the unselected basal population (G0). Wild radish adaptation in flowering time was also accompanied by changes in plant size (biomass and height), reflecting similar physiological responses in *A. thaliana* L. (Tienderen et al. 1996). In this study, the height of the first flower and vegetative above‐ground biomass were consistently reduced with early‐flowering time selection. As a result of insufficient biomass accumulation prior to flowering, early‐FT‐selected populations grow in a more prostrate form, lacking the ability to support reproductive branches (Supporting information). Given that in annual species like wild radish plant size is a predictive value of the amount of resources to be allocated to reproductive fitness (Weiner 2004; Weiner et al. 2009), the reduced biomass observed in early‐flowering plants is likely to cause reductions in the population's competitive ability for resources (Goldberg 1990) and overall fitness. Whilst not measured in this study, early‐FT‐selected plants are likely to have lower fecundity (Cheam 1986; Conner and Via 1993), due to lower biomass plants producing fewer and smaller flowers that produce less seeds per silique (Conner and Via 1993; Conner et al. 1996a,b; Williams and Conner 2001).

Despite this likely fitness cost, it is anticipated that early‐flowering plants will also have a fitness advantage whilst harvest weed seed control selection of retained seeds at crop maturity is occurring (Vila‐Aiub et al. 2009). The rate of fruit abscission was not determined in this study; however, as a consequence of early flowering time, the number of individuals in the population carrying well‐matured pods at the time of crop maturity would rapidly increase as flowering time is reduced. This increase in the proportion of well‐matured pods at harvest is expected to increase the probability of silique abscission prior to harvest, especially during periods of water deficit, high temperature or wind (Taghizadeh et al. 2012). However, seed retention amongst early‐flowering time‐selected populations is speculated to vary according to climatic conditions, which is likely to vary across differing agro‐ecosystems (Taghizadeh et al. 2012). Similarly, the reduction in wild radish flowering height and the resultant prostrate growth habit (associated with low biomass accumulation) is likely to reduce seed interception at the time of crop harvest as a greater proportion of siliques are likely to be located on unsupported stems, below the required height for harvest interception (Supporting information).

Within an agricultural context where weed species are selected with harvest weed seed control, the evolution of early‐maturing ecotypes can be seen as an optimal survival strategy. However, the presence of a steep fitness gradient favouring late‐flowering ecotypes implies that early‐flowering time‐selected populations are likely to rapidly moderate flowering time back to an ecological optimum when selection is relaxed, as later‐flowering ecotypes are likely to have a greater reproductive capacity (Baker 1974; Conner et al. 1996b). From a weed management perspective, in order to maintain long‐term effectiveness of harvest weed seed control techniques, it may be prudent to periodically stop the use of this selective tool once weed seed banks have been reduced to manageable levels. Any relaxation in selection is expected to allow for the recovery of the standing genetic variation in flowering time traits within wild radish populations, therefore restoring phenological traits that are important for the interception of weed seeds at harvest.

Our results also demonstrate the evolutionary capacity of wild radish populations to rapidly adapt when late‐flowering individuals are favoured. Within agro‐ecosystems, other human‐mediated selective tools that target early‐maturing phenotypes are used. Seed set reduction techniques such as herbicidal flower sterilization (termed as crop‐topping) (Madafiglio et al. 2006) target early‐flowering individuals before the maturity of the crop, effectively favouring the proliferation of late‐maturing phenotypes. Three generations of late‐FT selection resulted in a doubling of FT along with a 2.2‐fold increase in biomass and a 1.6‐fold increase in plant height at anthesis. However, FT adaptation favouring lateness is likely to be moderated by moisture stress during reproduction in Australia, reducing the fitness of these extremely late‐flowering ecotypes. Therefore, it is again speculated that the evolutionary effect of late‐FT selection would vary according to the climatic conditions to be more likely in agro‐ecosystems of higher rainfall and a longer growing season (Conner et al. 1996a).

The significant reductions and delays in wild radish flowering time as a result of FT selection further highlight the genetic potential of wild radish populations for rapid adaptive evolution in response to selection agents that act both prior and at the time of crop harvest.

## Data archiving statement

Data available from the Dryad Digital Repository: http://dx.doi.org/10.5061/dryad.3j380

## Supporting information


**Table S1.** Parameter estimates (days to flowering) following early‐flowering time selection using the four‐parameter logistic model [1] used to estimate FD_50_ parameters.
**Table S2.** Parameter estimates for the days to flowering following late‐flowering selection using the four‐parameter logistic model [1] used to estimate FD_50_ parameters.
