# Supplementary material for: Directional selection for flowering time leads to adaptive evolution in Raphanus raphanistrum (Wild radish)
Source: Evol Appl. 2016 Feb 17;9(4):619–29. doi: 10.1111/eva.12350 (PMC4831463; doi:10.1111/eva.12350)
Supplement: Supplementary file 1 — Table S1. Parameter estimates (days to flowering) following early‐flowering time selection using the four‐parameter logistic model [1] used to estimate FD50 parameters. Table S2. Parameter estimates for the days to flowering following late‐flowering selection using the four‐parameter logistic model [1] used to estimate FD50 parameters. [file EVA-9-619-s001.docx]

**Table S1.** Parameter estimates (days to flowering) following early flowering time selection using the four-parameter logistic model [1] used to estimate FD_50_ parameters. Standard errors for parameter estimates are in parentheses. Selection ratios were calculated based on FD_50_ values for the unselected commencing population (G0) and respective selected or unselected control progeny.

|  | **Population** | **d** | **b** | **e FD_50_^B^** | **e FD_50_ selection ratio** | **P value^A^** | **Shift from G0 (days; FD_50_)** | **First flowering individual** | **Flowering range (days)** |
| --- | --- | --- | --- | --- | --- | --- | --- | --- | --- |
| **Base** | G0 | 100 | –9.79 (0.26) | 59 (0.2) efg | – | – |  | 38 | 52 |
| **Selected** | EF1 | 100 | –9.65 (0.26) | 51 (0.1) d | 0.8 | <0.05 | –8 | 36 | 35 |
|  | EF2 | 100 | –19.19 (0.57) | 57 (0.1) e | 0.9 | <0.05 | –2 | 35 | 31 |
|  | EF3 | 100 | –11.70 (0.31) | 45 (0.1) c | 0.7 | <0.05 | –14 | 35 | 19 |
|  | EF4 | 100 | –11.38 (0.38) | 37 (0.1) b | 0.6 | <0.05 | –22 | 31 | 19 |
|  | EF5 | 100 | –14.38 (0.42) | 29 (0.1) a | 0.5 | <0.05 | –30 | 27 | 13 |
| **Unselected** | CE1 | 100 | –16.93 (0.57) | 61 (0.1) fg | 1.0 | <0.05 | 2 | 42 | 34 |
|  | CE2 | 100 | –10.54 (0.29) | 57 (0.1) eg | 1.0 | <0.05 | –2 | 42 | 32 |
|  | CE3 | 100 | –11.10 (0.30) | 59 (0.1) efg | 1.0 | 0.42 | 0 | 41 | 37 |
|  | CE4 | 100 | –9.96 (0.29) | 58 (0.1) ef | 1.0 | 0.37 | –1 | 36 | 45 |
|  | CE5 | 100 | –12.63 (0.41) | 62 (0.1) g | 1.0 | <0.05 | 3 | 41 | 41 |

^A^ LD_50_ P value comparing the difference between selected and commencing populations assessed by the SI function in the DRC package in R. v2.14.1.

^B^ FD_50_ parameters separated using Tukey’s protected LSD at the 5% level of significance.

Lack-of-fit P value for appropriateness of the four-parameter logistic model [1] – 1.0.

FD_50_ (Days after emergence)

**Table S2.** Parameter estimates for the days to flowering following late-flowering selection using the four-parameter logistic model [1] used to estimate FD_50_ parameters. Standard errors for parameter estimates are in parentheses. Selection ratios were calculated based on FD_50_ values for the unselected commencing population (G0) and respective selected or unselected control progeny.

|  | **Population** | **d** | **b** | **e FD_50_ ^B^** | **e FD_50_ selection ratio** | **P value^A^** | **Shift from G0 (days; FD_50_)** | **First flower** | **Flowering range (days)** |
| --- | --- | --- | --- | --- | --- | --- | --- | --- | --- |
| **Base** | G0 | 100 | –9.79 (0.26) | 59 (0.2) a | – | – |  | 38 | 52 |
| **Selected** | LF1 | 100 | –13.56 (0.44) | 81 (0.2) b | 1.3 | <0.05 | 22 | 61 | 41 |
|  | LF2 | 100 | –10.15 (0.30) | 80 (0.2) b | 1.3 | <0.05 | 21 | 59 | 48 |
|  | LF3 | 86 | –9.98 (0.34) | 114 (0.3) c | 1.9 | <0.05 | 55 | 52 | 84 |
| **Unselected** | CL1 | 100 | –11.86 (0.34) | 60 (0.1) a | 1.0 | 0.26 | 1 | 39 | 42 |
|  | CL2 | 100 | –14.04 (0.44) | 63 (0.1) a | 1.1 | <0.05 | 4 | 47 | 34 |
|  | CL3 | 100 | –13.98 (0.42) | 60 (0.1) a | 1.0 | 0.14 | 1 | 46 | 32 |

^A^ LD_50_ P value comparing the difference between selected and commencing populations assessed by the SI function in the DRC package in R. v2.14.1.

^B^ FD_50_ parameters separated using Tukey’s protected LSD at the 5% level of significance.

Lack-of-fit P value for appropriateness of the three parameter logistic model [1] – 1.0.

FD_50_ (Days after emergence)
